# Observations on Functional Disorders of the Spinal Cord, and Their Connexion with Hysterical, Nervous, and Other Diseases. Illustrated by Cases, Selected Chiefly from the Reports of the Pallas, Kenry, and Currah Dispensaries.

**Published:** 1829-12

**Authors:** Wm. Griffin, D. Griffin

**Affiliations:** Limerick.; Limerick.


THE LONDON
### Medical and Physical Journal.

No. 370, VOL. LXII.] DECEMBER, 1829. [No. 42, *New Series.*

For many fortunate discoveries in medicine, and for the detection of numerous errors, the world is indebted to the rapid circulation of Monthly Journals; and there never existed any work, to which the Faculty, in Europe and America, were under deeper obligations than to the *Medical and Physical Journal of London*, now forming a long but an invaluable series.—*Rush.*

### ORIGINAL PAPERS, AND CASES,

### OBTAINED FROM PUBLIC INSTITUTIONS AND OTHER AUTHENTIC SOURCES.

### FUNCTIONAL DISORDERS OF THE SPINAL CORD.

*Observations on Functional Disorders of the Spinal Cord, and their Connexion with Hysterical, Nervous, and other Diseases. Illustrated by Cases, selected chiefly from the Reports of the Pallas, Kenry, and Currah Dispensaries.* By Wm. Griffin, M.D. and D. Griffin, M.R.C.S., Limerick.

Although the attention of the medical profession has been, within these few years, so very much directed to affections of the spinal cord, it would seem, on inquiry, that little really new has been added to our previous acquisitions on the subject. Referring to many valuable opinions of German and French writers, long fallen into undeserved neglect, and comparing them with inferences now almost obtruding themselves on general notice, we shall be apt to imagine there has been, in this country at least, an unaccountable carelessness of observation; or be led to very unsatisfactory reflections on the impenetrable obscurity in which all disorders of the nervous system would seem to be involved. Perhaps it is because of the little fruit gathered by long toil, that some are inclined to consider the labour misapplied; and while they are satisfied to treat, under the appellation of nervous, hysterical, &c., whole trains of complaints which have little in common but that of their being ill understood, venture to censure the persevering efforts of those who, if they have been as unsuccessful, have at least the merit of not being so hopeless, in the inquiry. It is gratifying to observe that the rapid progress which modern physiology is making, daily exposes the futility of opinions which measure the value of application only by its ill success in particular instances, and offers new proof that the worth of a discovery in obscure sciences is proportioned almost as much to the expenditure of time and thought as to the strength of the intellect which has made it.

But, exclusive of these considerations, and even of the temptation to new exertion in the late extraordinary discoveries elicited by the labours of Magendie, Le Gallois, Wilson Philip, and Charles Bell, the subject of spinal disease holds out a most imperative inducement, in the perplexed state of our diagnosis, through a vast range of complaints, and in the acknowledged mistakes which we every day see made in the practice of well-informed members of the profession. We need only refer to the periodicals of the day, which teem with cases of, as they are called, strange, anomalous, proteiform maladies, the mocking birds of nosology, or imitators of every known disease, accompanied with cautions on the danger of confounding them with their prototypes: or to the admitted difficulty in the treatises on nervous and hysteric diseases, by the most distinguished practitioners of the day, of offering any marked symptoms by which they could always be clearly distinguished.* Indeed, the actual existence of these

* Dr. Hamilton, in his Treatise on Diseases of Women and Children, says, " On some occasions, hysterics put on the appearance of several disorders, such as melancholy, epilepsy, palsy, inflammation of the lungs or bowels, gravel, &c. It requires in these cases not only the *most unremitting attention*, but also the *utmost practical discernment*, to distinguish the true disease from that which it resembles.

"When the symptoms are not uniformly and regularly those which occur in the ordinary cases of the disease imitated ; when there suddenly seems great danger, without those previous changes in the progress of the complaint which are usually met with; when there is either a natural state of the pulse with alarming symptoms, or a very frequent irregular pulse without any affection of the breathing or shrinking of the features, there is reason to suspect hysterics as the true disorder. *Cases from time to time occur where it is impossible to ascertain the real nature of the affection, till towards its termination.* The fact, too, that in every acute disease of women which requires copious evacuations, or which debilitates the system, hysterics are apt to occur in the progress to recovery, adds much to the difficulty of judging precisely in any given case."

Dr. Gregory, in his Practice of Physic, after mentioning the frequent resemblance and connexion between hysteria and epilepsy, and the difficulty of distinguishing one from the other, says, " But it is not only from epilepsy that hysteria is difficultly distinguished. There is hardly a disease in the whole nosology of which it has not imitated the symptoms, and that with surprising accuracy. I have seen hysteria accompanied by constant vomiting; by a complete ischuria renalis; by the most obstinate colic ; by all the symptoms of genuine asthma. Authors have described in like manner an hysterical jaundice, an hysterical mania, an hysterical diabetes. These circumstances require to be borne in mind with reference to prognosis."

singular and apparently functional disorders, which assume the symptoms of all others, and continue unrelieved by the remedies applicable to any; which look like inflammations, and are rendered worse by bloodletting; or simulate intense spasmodic attacks, and bid defiance to opiates; and which yet, after resisting all possible treatment, eventually, and perhaps suddenly, disappear of themselves, would, in a merely therapeutic view, appear strange and deserving of inquiry.

In the classification of diseases, the great division into those of the vascular and nervous systems, the pyrexiæ and neuroses of Cullen, was at once obvious to the nosologist; and, again, the distinction of nervous complaints, as they developed themselves in any one tissue or organ, or another, and invariably presented the same characters, seemed easy of attainment; but there came to be a great difficulty about that vast number of the latter which are so variable in their seat and appearance, and so untraceable in their origin, as apparently to preclude all proper arrangement. They were necessarily thrown together as a sort of anomalous class, like the Cryptogamia in botany, awaiting the result of future discovery for more appropriate distribution. But, surely, if it had been considered that all nervous disorders must eventually resolve themselves into affections of the three great centres of nervous influence, the cerebral, the spinal, and ganglionic systems, much of this perplexity might have been avoided. We have, it may be presumed, now made sufficient progress in the pathology and diagnosis of diseases of the brain to estimate with some accuracy the characters which indicate their origin; and may generally, even in those that most nearly resemble spinal or other affections, draw correct inferences from the history of the case and the intensity of particular symptoms. Of those of which there is much doubt, or which evidently do not belong to the brain, we can observe how far they correspond with the usual or known symptoms of spinal disorder, organic or functional; and, if there be some found bearing no analogy to either the one or the other, the deduction seems clear that they depend on some disordered or diseased state of the ganglionic system. However hypothetical a division of this kind may appear in diseases of the nervous tissues, which are so delusive and difficult of arrangement, and after death present so few appearances to direct the reasonings of the pathologist; however frequently erroneous its application may prove, it must be true in principle, and, when once held in view, cannot but give more of method and object to our investigations, and greater rationality to our treatment.

The cerebral, the spinal, and ganglionic systems are all, independently of one another, though perhaps unequally, subject to inflammation, to irritation, and the influence of sedative powers. The spinal cord itself, the experiments of Le Gallois have shown, is composed of portions independent of one another in their powers and functions, being centres from which the nervous actions of corresponding parts of the body emanate, and to which they tend. At least four of the senses derive their faculties from its superior portion,* which can receive their usual impressions and originate actions independent of the brain or cerebellum. Its anterior part is the source of all voluntary motion, its posterior of all common sensation. Between both is the column from which arises Mr. Bell's respiratory system of nerves, the most susceptible and independent of all, and yet we find a perpetual disposition to attribute all nervous diseases to affections of the brain, as if it were the sole sentient organ and seat of universal sympathy.

That a vast proportion of those diseases may be attributed to irritation at the origin of the spinal nerves, without any cerebral affection, was asserted by Ludwig. He even explained the phenomena of many hysterical affections by the connexion of these nerves with the par vagum. Other eminent men have held the same opinions. Mr. Burns, of Glasgow, in his work on Midwifery, has given a very excellent and interesting chapter on spinal irritation, evidently the result of long experience and acute observation, in which he asserts that its visible consequences are so various it is impossible to classify them. In a late Number of the Glasgow Medical Journal, Dr. Brown has published an essay pointing out the connexion between it and many painful affections, usually treated as rheumatic or disorders of the viscera. He has cited some valuable cases, and offered much ingenious reasoning on the causes to which they may in general be probably attributed. How very little these opinions have as yet influenced the science, any one acquainted with modern medical literature or medical practice must be aware.

It is surprising when a subject is once universally admitted to be obscure and perplexing, with what little scrutiny or hesitation we receive any name or phrase that relieves us from the impression of total ignorance. To this alone

* The spinal cord is always mentioned as including the medulla oblongata.

can be attributed the ready acceptance of the words imitation, proteian, anomalous, &c., as applied to hysteric and nervous diseases, as if there existed in the animal economy some evil influence, without home, or habit, or relation, capable of increasing or interrupting any of its functions, or assuming any of its morbid actions, yet free and independent of all organic change. The convenience of referring to such influence all the morbid phenomena which are difficult of explanation, bears a just proportion to its mysterious nature; but surely we might as well speak of labour imitating cramp, as of hysteria imitating croup, the one a spasmodic affection of the gastrocnemii muscles, occasioned by pressure or irritation of the sacral nerves, the other a spasmodic affection of the muscles of the larynx, occasioned by irritation of the cervical.*

It is not to be inferred from these remarks that any thing approaching certainty as to the nature of those numerous diseases can be yet attained. We only contend against that catching, unconscious indolence which leads us to prefer vague modes of expression to active inquiry. Accurate observation and industrious research are never wholly useless, even when they fail in establishing the inductions sought for; and this conviction, perhaps as much as the hope of unravelling those intricate affections, induced a perseverance in investigations which the present paper will show were not without considerable trouble. Its object is chiefly to illustrate their apparent connexion with a morbid state of the spine, and to point out the immense proportion they bear among the complaints of young females, beyond what has been generally imagined. Remarkable instances will be given of the inutility of all symptomatic treatment in some of them, and the successful, and sometimes almost magical, influence of the remedial means, when once directed to the spine. The successful cases were not, it must be admitted, of the most complicate class, but they were such as, when not understood, always prove sufficiently obstinate to weary the patient and embarrass the practitioner. Of the more perplexing ones, where general irritation of the spinal column prevailed, it is to be regretted the practice of two dispensaries, at which 4000 patients were annually attended, afforded a more ample opportunity of studying the history and character,

* If we were to inquire, in a case of labour, what is this spasmodic and painful affection of the gastrocnemii? and it was answered, It is not idiopathic cramp, but an affection exactly resembling it, dependent on the parturient state, one, in fact, of the many anomalous complaints which labour is found to imitate, would it be conceived in the slightest degree satisfactory?

than of discovering much that was new in the management. If, however, to become acquainted with all the possible relations of a disease, and to approach nearer to its probable origin, be any advance towards an improved plan of cure, even what has been done may not be considered unimportant.

Perhaps the extraordinary case which first arrested the notice of the writers of this article, and directed their attention especially to disorders of the nervous system, may also prove its most interesting introduction to the reader.

I. A young lady, aged twenty-one, who had always before enjoyed good health, received a slight blow on the chest from her mother, during her convulsive struggles while dying of apoplexy. She spit up a little blood at the time, and felt pain for some days; after which it suddenly removed to the abdomen, affecting the left side, about the situation of the descending colon, with frequent hard pulse and tenderness, and the most incessant vomiting. The pain was abated by bleeding, blistering, and aperients; but nothing could allay the vomiting, which was brought on by the smallest quantity of any thing, solid or liquid, taken into the stomach. This came to be attended with flitting pains in the head, with throbbing of the temples, and intolerance of light, attributed to the straining; the continuance of which made it difficult to move the bowels. Even when medicine did operate, it gave no relief.

She remained many days in this state, suffering much from want of rest and the distressing retching; after which she was attacked with frequent oppression, occurring at intervals through the day, and usually terminating in fits of insensibility. In these she usually lay for ten or fifteen minutes, with her hands fast clenched, or sometimes shutting and opening them alternately with great rapidity. There was considerable rigidity of the tendons of the wrist, while the fit lasted; and the first symptom of amendment was always a gradual relaxation and opening of the fingers, when she fetched a long deep sigh, and recovered.

These oppressions proved as intractable as the vomiting, and were very distressing. Repeated blistering, ether, assafœtida, opium, and other antispasmodics, were had recourse to without relief, except of the most temporary kind. At the end of three weeks, however, the more severe symptoms of the complaint, without any very obvious cause, and after resisting every kind of treatment, began gradually to decline: the oppressions, throbbing at the temples, fits of insensibility and vomiting, manifestly abated; and the digestive organs, the state of which had never been lost sight of, improved rapidly under mild aperients and bitters. In short, she soon after recovered a sufficient degree of health to permit her going to a party, and even joining in the amusements.

This reprieve was but of very short continuance. A return of the oppression brought with it cough, pain in the chest and left side; the former slowly disappearing as the latter symptoms advanced and became more formidable. The cough was loud, dry, and convulsive, and became at last so incessant that she had no intermission of the fits by day or by night. The convulsive expirations followed one another with such rapidity, that they can only conceive the suffering who have witnessed a severe chincough, and imagine the fits following one another without interval. To heighten the distress, it increased considerably the pain in the chest and sides, and the respiratory muscles became so sore and tender, from the eternal convulsive action, that she could scarcely bear to have a finger touch them. After much time had passed in vain attempts to remove or alleviate it, she became affected with swelling and pain in the anterior part of the right lobe of the liver, which increased rapidly, and formed a round, circumscribed, shining tumor, bearing all the appearance of an abscess. This was very painful, and the torture produced by the constant coughing was extreme.

A course of blue pill was now prescribed at a consultation; the symptoms, and especially the cough, being attributed to an affection of liver, which was supposed to have existed for a long time, although only now developing itself. Copious ptyalism followed, and, to the great gratification of every one, the cough was now first relieved, and in a week or two ceased altogether.

The young lady, however, remained in a very weak, complaining state, troubled much with occasional pain in the head and intolerance of light, and eventually, as the soreness of the gums diminished, the terrific cough again evinced a disposition to return. It was not considered advisable to persevere in the mercurial pill, which seemed to be the only preventive likely to be employed with success, as she had suffered much from the salivation, and was greatly debilitated. The consequence was a renewal of her sufferings, if possible, to a more intense degree than before. New symptoms week after week supervened, or alternated with the old, and were only more distressing on

2

account of their strangeness and suddenness of attack: at one time the oppressions; at another, headach with fits of insensibility; at a third, the old pains traversing different parts of the colon and ileum with their former violence. She was attacked, too, with severe pain and tenderness in the hypogastric region, followed by retention of urine, obliging the introduction of the catheter. But little was drawn off, however, as the secretion was almost entirely suppressed, and did not return for three or four days, when the soreness, &c. in the hypogastric region subsided. During all this time the pain and tenderness of chest, and the dry, loud cough, were never for a moment absent.

The case was now looked upon as quite hopeless: the distress occasioned by such complicated disorder destroyed all rest and appetite, and induced extreme emaciation; solid food could no longer be borne, but was either instantly rejected, or excited violent spasmodic pain in the stomach, and sometimes the oppressions. The slightest motion (she was now continually confined to bed,) brought on similar paroxysms, after which she usually became almost insensible, with suppressed convulsive efforts at coughing, her voice gone, and her pulse rapid. This state generally lasted for some hours, sometimes much longer; and, as strength gradually returned, the hacking eternal cough resumed its attack.

It would be tedious to enter into a minute history of the symptoms or treatment during the succeeding two or three years. The disease successively assumed the appearance of organic disease of the lungs, heart, and abdominal viscera, and, though the sufferings of the young lady may be supposed to have diminished little, she continued to live, and apparently to maintain the little strength the earlier attacks had left to her. She lived almost entirely on milk, and of this not more than half a pint was taken in the day. A small portion of ripe fruit, a strawberry or cherry, was taken occasionally in the summer-time, and sometimes a little jelly in the winter. Little medical treatment was made use of, except some attention on the part of her friends to her general health, and occasional attempts at relieving particular symptoms by opiates, antispasmodics, or blisters.

On an accidental visit of her medical attendant at the close of the year 1828, the connexion between several of the pains of which she complained and the distribution of the spinal nerves appeared so striking, that an examination of the spine was made. There was no deformity, unevenness, or prominence of the vertebræ, but extreme tenderness of the whole column. Pressure on any of the spinous processes excited instant convulsive fits of coughing, and pain at the corresponding point anteriorly, or oppression. The slightest curvature in any direction was intensely painful; attempting to turn in the bed during the examination (which, however, she could never either accomplish or permit,) occasioned a sensation as if her back was breaking; raising the head from the pillow, and bending the neck forward, brought on a burning pain at the middle dorsal vertebræ, which shot down to the extremity of the spine, and thence to the limbs, knees, and toes, followed by a sort of general cramp. It seemed extraordinary how little the patient directed attention to the back in so intense a case of spinal disease: she frequently complained of pain there; but, as it was never constant like those felt at the extremities of the nerves, and was only excited by pressure or motion of the spine, and was then generally accompanied by, or occasioned, extreme sickness of stomach, retching, and eventual insensibility, it claimed little notice in the train of symptoms.

The complaint now clearly developed itself. The various affections to which she had been so long a sufferer were obviously attributable to some disease of the medullary column. The distressing headach, rushing of blood to the head, ringing in the ears, throbbing at the temples, and fits of insensibility; the sensation of acute pain, or of the pricking of pins and needles, shooting forward through the face and jaws, in the course of the branches of the fifth pair of nerves, or lower down in front of the larynx; the difficulty of swallowing; the shrill croupy breathing; the pain and cramp of the stomach or chest; the oppression, and the dry, loud, convulsive cough, were all readily referred to disease or irritation of the cervical portion of the spinal cord. The extreme soreness and pain of chest and sides; the pain at the upper part of the sternum, shooting down the arms to the fingers, and producing distressing tingling; the occasional numbness of the arms; the symptoms of cardiac and pulmonic disease, appeared to depend upon some affection of the upper dorsal and lower cervical: and the abdominal pain; tendinous cramp; colic, and sometimes seeming inflammatory attacks; and those of dysury, or total suppression of urine, or painful affections of the limbs, were at once traced to some altered state of the lumbar and lower dorsal portion. All the complicated, and it would appear whimsical, attacks of this strange malady seemed now simple and necessary results, and their alternations with one another merely indicated the shifting of the diseased action to new points of the vertebral chain.

As issues, or blisters to the spine, were almost the only untried remedies which the state of the patient suggested, and these seemed wholly inadmissible, from the difficulty and pain with which the slightest motion of the frame was attended; as it appeared also possible that sloughing or gangrene might take place in so emaciated a person, the case was again left to the efforts of nature; care, however, being taken of the state of the bowels; and narcotics, &c. resorted to, as before, when in violent pain. The disease was, nevertheless, slowly progressive, and as it advanced declared its true seat to the most careless observer: the whole spinal column was, if possible, more acutely tender; the slightest pressure or motion brought on pain; cramps, or fits of retching; drawing the sheet or arranging the bed, or the sudden falling of a piece of furniture, excited an instant paroxysm, commencing with cramp in the chest; sense of suffocation in the throat, with low crowing inspiration, not ringing and stridulous as in croup; and terminating in extreme debility, with total loss of power, and tremulous convulsive motion of almost every muscle in the frame. The affection of head and pain in the throat became more tormenting; there was constant distressing pain of stomach, with rawness, soreness, and sometimes a burning feel, extending up the trachea to the larynx; there was variable pain of the chest and left side, and a sensation as of a sore cord or band stretched across from the superior bone of the sternum to a point corresponding with the anterior part of the fifth rib on the left side. This never permitted her stretching back (making the chest prominent), and she had often apprehensions that it would rend or snap in the violent fits of coughing. She had also a frequent feeling as if the spine was seized internally, and drawn to the sternum or stomach: when to the former, the sensation was succeeded by convulsive spasms, with oppression; when to the latter, by violent cramp extending upwards to the sternum, and shooting down to the limbs, knees, and toes. At times, when the cough was extremely violent, and shook the frame much, or when the patient was lifted on a sheet to have her bed arranged, she felt as if the articulating surfaces of the spinal bones were inflamed, sore, and glided or rubbed upon one another in the loose ligaments. This feeling was so excruciating, that, whenever she was about to be removed on a sheet, she was accustomed to throw all the extensor spinal muscles into action, and, by a violent effort, bring the whole spine into a state of rigid extension, to preclude the possibility of the slightest motion. An approach to syncope always followed the exertion, in which she lay on the bed for days, unable to speak or swallow, or even move, yet conscious of every thing passing about her. Although so seemingly still and breathless that it might have been imagined she lay in an utter state of relaxation and exhaustion on these occasions, if a hand was laid on hers, it was found in rigid spastic action, and, instead of reposing quietly on the chest as it appeared, pressed firmly and almost convulsively against it, as one does to prevent the elevation of the ribs in painful breathing. The breathing, too, although so apparently easy as to be almost imperceptible, was found, on close observation, difficult and suffocating; there was a subdued working of the muscles of the throat, and inspiration was either wholly suspended at times or occurred in short indistinguishable catches, until a deep sigh brought with it general relaxation and relief. It was usually a full week before she recovered from the ill effects of these attempts to move her from the bed; but even turning her head on the pillow for a few minutes brought on such convulsive coughing, and subsequent sinking, that she could not utter an audible whisper, and would lie for hours in a state of the most extreme exhaustion.

As it seemed that her sufferings could now, at all events, admit of little increase, an issue was inserted at each side of the second cervical vertebra; by which the pain of the forehead, face, and scalp was considerably relieved; all the parts, as she said herself, above the issue were better, the other symptoms were little altered.

Towards the close of February 1829, while drinking in the evening, she felt a sensation as if something gave way in her chest, as if the band from the upper part of the sternum, before spoken of, had snapt. She was instantly attacked with oppression, a sense of burning and pain in the throat and chest, croupy breathing, total loss of speech, and blindness of the left eye, with numbness and paralysis of the left arm; she had also a sense of numbness extending from the point in the chest where she felt the band snap, across to the shoulder, and down the left arm to the fingers; some difficulty of swallowing, and violent pain, straining, or retching, when the smallest quantity of food or drink reached the stomach. There was some swelling and excessive tenderness of stomach, with violent cramp at intervals, which extended down to the limbs and knees. The secretion of urine was suppressed, no more than half an ounce having passed in twenty-four hours, and that thick and black. There was no tenderness or fulness in the pubic region.

After the lapse of some days, during which croton oil and diuretics had been freely used, the eye partly recovered its power, and the action of the kidneys was restored. Blisters to the throat and neck were of little advantage; but, on applying one to the occiput, some degree of voice was manifestly recovered, and the power of swallowing perfectly; the fingers of the paralysed arm also seemed to acquire a little motion.* In July, a very decided improvement had taken place. The arm had attained much strength; and she was able to speak in a low whisper, though with pain and difficulty. It should be observed, that the power of articulating was never lost, so that, even while partly dumb, she could often make herself understood by a distinct, voiceless articulation of the words.

We have at length brought the history of this melancholy complaint down to the present moment, and venture to express a hope we shall hereafter have to record its favorable termination. After all the young lady's sufferings, there is no evident sign of any irremediable mischief having occurred; and, however small the quantity of nutriment she is able to take, the expenditure of power has become so accurately proportioned to it, that we may suppose little need be apprehended from debility. In conclusion, perhaps, there is yet one circumstance worth mentioning: the singular change which, in the course of the complaint, took place in those fits of insensibility, or powerlessness, which were said to approach a state of syncope. In the commencement they very closely resembled slight tetanic paroxysms, during which there was a degree of consciousness to all that was passing around her; they then succeeded the rushing of blood to the head, &c., and she lay staring with a wild glassy look on all about, without power of speech or motion; but latterly they came on like cataleptic trances, fixing her, like a waxen figure, in whatever position she chanced to lie, for ten or twenty minutes, or longer.

In a late visit to the patient, it was gratifying to observe the amendment. She now speaks perfectly well, is cheer-

* The paralytic attack seemed in the first instance to have affected the whole side; for, although she never complained of the left leg, it was observed, in those convulsive thrillings of the frame which succeeded paroxysms of the pain and oppression, to remain perfectly still. It continued, however, capable of the usual voluntary motions.

ful, and entertains hopes of recovery. She complains, however, that, instead of the tight sore band across the chest, (the snapping of which, though attended by such extraordinary symptoms, gave her great relief,) she now feels a sore tumor as if growing from the spine, and hitting against the sternum in front every time she coughs. Can these sensations really have a connexion with any such affections as she conjectures? Was the snapping of the band the rupture of an adhesion, and why the paralysis? These are interesting questions. She mentions that, for days before the presumed lesion took place, she felt the band giving more and more with the fits of coughing; she felt it tearing, and, after the complete rupture, floating loose in the chest.

[To be continued.] Vol. LXIII. p. 13.

---

